# DEL in China: the D antigen among serologic RhD-negative individuals

**DOI:** 10.1186/s12967-021-03116-6

**Published:** 2021-10-20

**Authors:** Qinan Yin, Willy Albert Flegel

**Affiliations:** 1 Laboratory Services Section, Department of Transfusion Medicine, NIH Clinical Center, National Institutes of Health, Bethesda, MD 20892 USA; 2 Henan University of Science and Technology, Luoyang, Henan China; 3 Huazhong University of Science and Technology, Wuhan, Hubei China

**Keywords:** Rh blood group, RhD-negative, Ethnicity, Pregnancy, Transfusion, RhIG

## Abstract

**Background:**

Providing RhD-negative red cell transfusions is a challenge in East Asia, represented by China, Korea, and Japan, where the frequency of RhD-negative is the lowest in the world.

**Findings:**

Among 56 ethnic groups in China, the RhD-negative frequency in Han, the prevalent ethnicity, is 0.5% or less, similar to most other ethnic groups. The Uyghur ethnic group has the highest reported RhD-negative frequency of up to 4.7%, as compared to 13.9% in the US. However, an estimated 7.15 million RhD-negative people live in China. The RhD-negative phenotype typically results from a loss of the entire *RHD* gene, causing the lack of the RhD protein and D antigen. The DEL phenotype carries a low amount of the D antigen and types as RhD-negative in routine serology. The DEL prevalence in RhD-negative individuals averages 23.3% in the Han, 17% in the Hui and 2.4% in the Uyghur ethnicities. The Asian type *DEL*, also known as RHD*DEL1 and *RHD:c.1227G* > *A* allele, is by far the most prevalent among the 13 *DEL* alleles observed in China.

**Conclusion:**

The purpose of this review is to summarize the data on DEL and to provide a basis for practical strategy decisions in managing patients and donors with *DEL* alleles in East Asia using molecular assays.

## Introduction

The transfusion of RhD-negative red cells, often in short supply, poses a particular challenge in East Asia [1, 2]. The lowest frequency of RhD-negative in the world is found in China, Korea and Japan [2, 3]. The first Rh typing for mainland China occurred in 1949 [4], Taiwan in 1956 [5], Macao in 1959 [6] and Hong Kong in 1961 [7], with reported RhD-negative frequencies of 1.92% or less. Even some of these low rates have overestimated the true frequency of RhD-negative, as the quality of the anti-D reagent may have varied. The RhD-negative frequencies are much greater in any of the other world populations, ranging from typically 10% in Africa, somewhat greater in North America, to up to 17% in Europe, where the greatest rate exceeding 20% occurs in the Basque population of Northern Spain.

The DEL phenotype is a D variant with a low number of D antigens per red cell [1, 8, 9]. Patients and donors with a DEL phenotype are routinely typed as RhD-negative in blood group serology, although they carry the D antigen. They should be considered RhD-positive for several clinically relevant applications [9–12]. DEL has been reviewed before [3, 13–15], albeit not with specific focus on East Asian populations.

### RhD-negative phenotype

#### Ethnicities in China

**Fig. 1:**
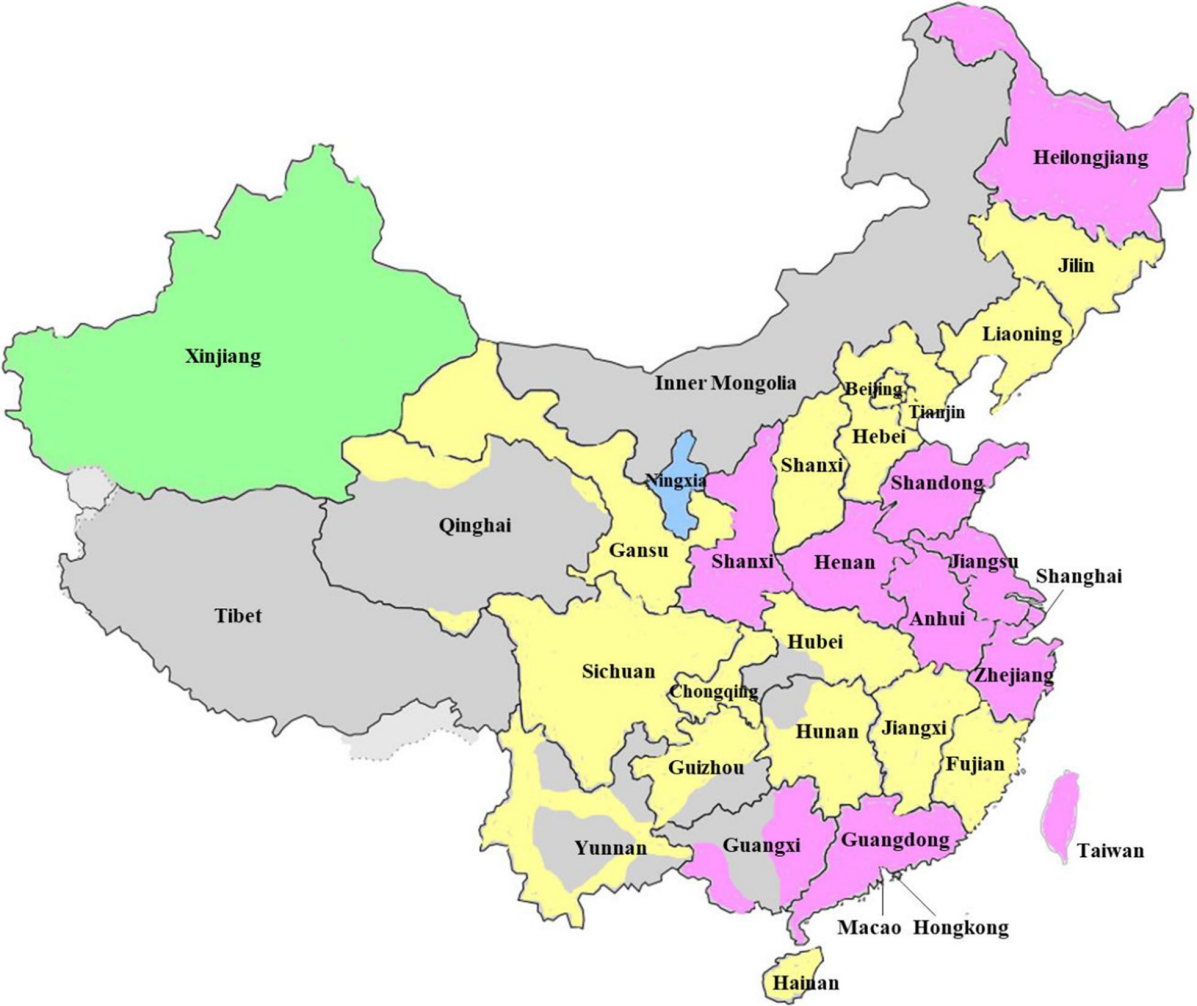
Distribution of ethnic populations in mainland China and Taiwan. The ethnic groups presented in this review are schematically depicted. The DEL phenotype or RHD*DEL1 genotype has been studied for Han, as the prevalent ethnicity, in many regions, provinces and cities (purple) but not in all (yellow). The data for Hui (blue) and Uyghur ethnicities (green) came from Ningxia and Xinjiang, respectively. No data on the DEL phenotype or RHD*DEL1 genotype is available for the remaining 53 ethnic populations in their typical regions of settlement (grey, see Table 1)

China recognizes 56 ethnic groups [16]. Among them, the Han group accounts for 91.13% of the whole population (Table 1) [17–25]. The Han group itself has a certain genetic diversity and substructure [26, 27]. Molecular data for the *RHD* gene among RhD-negative Han individuals have been established in various localities, but not in all provinces of China. A few results are known for only 2 other ethnic populations in China (Fig. 1).

**Table 1:** Estimate of RhD-negative among the 56 ethnic populations in China

Ethnicity	Population size^a^		RhD-negative individuals in the ethnicity		References
	n	%	Reported frequency	Estimate (n)	
Han	1,220,844,520	91.13%	0.24–0.50%	2.93–6.10 million	[17, 18]
Zhuang	16,926,381	1.26%	0.49%	82,939	[19]
Hui	10,586,087	0.79%	0.80–1%	84,689–105,860	[19, 20]
Manchu	10,387,958	0.78%	0.40%	41,552	[19]
Uyghur	10,069,346	0.75%	3.30–4.70%	332,288–473,259	[19, 21]
Miao	9,426,007	0.70%	0.70%	65,982	[19]
Yi	8,714,393	0.65%	1.30%	113,287	[19]
Tibetan	6,282,187	0.47%	0.60%	37,693	[22]
Mongolian	5,981,840	0.45%	0.30–0.50%	17,946–29,909	[19, 20]
Dong	2,879,974	0.21%	0.10%	2,880	[23]
Buyi	2,870,034	0.21%	0.40%	11,480	[23]
Kazakhs	1,462,588	0.11%	2.90%	42,415	[20]
Shui	411,847	0.03%	0.10%	412	[23]
Others^b^	32,881,690	2.45%	~ 0.10%	32,882	Estimate
Total	1,339,724,852	100%	N/A	3.80–7.15 million	N/A

*N/A* not applicable

^a^Calculation based on the 6th National Population Census of the People’s Republic of China of 2010 [24, 25]

^b^The other 43 ethnicities in order of population sizes (proportion) are the Tujia (0.63%), Yao (0.21%), Bai (0.15%), Korean (0.13%), Hani (0.12%), Li (0.11%), Dai (0.09%), She (0.05%), Lisu (0.05%), Dongxiang (0.05%), Gelao (0.04%), Lahu (0.04%), Wa (0.03%), Nakhi (0.02%), Qiang (0.02%), Tu (0.02%), Mulao (0.02%), Xibe (0.01%), Kyrgyz (0.01%), Jingpo (0.01%), Daur (0.01%), Salar (0.01%), Blang (0.01%), Maonan (0.01%), Tajik (< 0.01%), Pumi (< 0.01%), Achang (< 0.01%), Nu (< 0.01%), Evenki (< 0.01%), Gin (< 0.01%), Jino (< 0.01%), De’ang (< 0.01%), Bonan (< 0.01%), Russ (< 0.01%), Yugur (< 0.01%), Uzbek (< 0.01%), Monba (< 0.01%), Oroqen (< 0.01%), Derung (< 0.01%), Hezhen (< 0.01%), Gaoshan (< 0.01%), Lhoba (< 0.01%), and Tatars (< 0.01%)

The reported frequency of an RhD-negative phenotype in Han ranges from 0.24% to 0.50% (Table 2) [17, 18, 28–34]. Among a total of 1,929,664 Han individuals tested in 8 studies (Table 2), only 5771 carried the RhD-negative phenotype, resembling an average of 0.30%, similar to that of many other ethnic groups in China (Table 1).

**Table 2:** Prevalence of RhD-negative in Han

Regions	Province/city	Subjects		RhD-negative		References
		n	Type	n	Frequency^a^	
Eastern China	Shanghai	400,253	Donor	1585	0.40%	[28]
Southeastern China	Guangdong	41,905	Donor	102	0.24%	[17]
	Jiangsu, Guangdong, Guangxi	42,306	Patient	165	0.39%	[29]
Central China	Anhui	313,250	Patient	808	0.26%	[30]
	Anhui	30,799	Donor	155	0.50%	[18]
	Henan	38,526	Donor	106	0.28%	[31]
Western China	Shanxi	890,403	Donor	2385	0.27%	[32, 33]^b^
Hong Kong	N/A	172,222	Donor	465	0.27%	[34]
Total	N/A	1,929,664	N/A	5771	0.30%	N/A

*N/A* not applicable

^a^RhD-negative frequency among Han: 0.33% ± 0.09% (mean ± SD) for the 8 studies, 0.24–0.50% (range)

^b^Same dataset published in Chinese [32] and English [33]

The Uyghur group has the greatest reported RhD-negative frequency (3.3–4.7%) [19, 21], followed by the Kazakhs group (2.9%) [20], as compared to 13.9% in the USA [3]. The Uyghur and Kazakhs groups reside in the northwestern parts of China and likely descent from Central Asian ancestry, with more recent common ancestors with Caucasian ethnicities. This may explain their greater rates of RhD-negative phenotypes compared to ethnic groups further east and south.

Up to an estimated 7.15 million individuals in China are RhD-negative (Table 1).

#### RhD-negative, DEL and the *RHD* gene

More than 70% of the RhD-negative phenotypes in the Han ethnicity are explained by a loss of the entire *RHD* gene [35–37]. These individuals lack the RhD protein and have no D antigen. The remainder of the serologic RhD-negative Han individuals actually carry the *RHD* gene [9, 17]. Most of them express a DEL phenotype [9, 17]. They type as RhD-negative in serologic routine testing and, using serology, can only be detected by labor-intensive adsorption and elution techniques [8, 9, 12] or a limited association with distinct serologic Rh types [38]. We summarize published data that detection by molecular diagnostic is straightforward.

### DEL phenotype and alleles

#### Serologic DEL phenotype among Han

The DEL prevalence was first surveyed in 1993 in Hong Kong [34]. Since then, the DEL phenotype has been carefully documented in serologic RhD-negative Han using an adsorption-elution method [17, 18, 28–31, 39–47]. The reported frequency of the DEL phenotype in RhD-negative Han ranges from 16.3% to 32.6% (Table 3). Among a total of 6,470 RhD-negative individuals tested in 16 studies (Table 3), 1505 tested positive by adsorption-elution. Hence, an average 23.3% of RhD-negative Han individuals carry DEL phenotype. Up to an estimated 1,666,000 individuals are DEL positive in China. And 5.45 million lack the RhD protein, which puts them at risk of anti-D immunization, similar to RhD-negative Caucasians [48].

**Table 3:** DEL phenotype in serologic RhD-negative Han by adsorption-elution method

Regions	Province/city	RhD-negative individuals (n)	Adsorption-elution (n)		DEL phenotype frequency^a^	References
			Negative	Positive		
Eastern China	Shanghai	1585	1306	279	17.6%	[28]
	Shanghai	441	369	72	16.3%	[39]
	Shandong	74	52	22	29.7%	[40]
Northeastern China	Heilongjiang	374	312	62	16.6%	[41]
Southeastern China	Zhejiang, Jiangsu	643	488	155	24.1%	[42]
	Jiangsu, Guangdong, Guangxi	165	124	41	24.8%	[29]
	Guangdong	102	76	26	25.5%	[17]
Central China	Anhui	808	630	178	22.0%	[30]
	Anhui	515	373	142	27.6%	[43]
	Anhui	155	124	31	20.0%	[18]
	Henan	106	78	28	26.4%	[31]
Hong Kong	N/A	465	329	136	29.2%	[34]
Taiwan	N/A	395	269	126	31.9%	[44]
	N/A	294	200	94	32.0%	[45]
	N/A	230	155	75	32.6%	[46]
	N/A	118	80	38	32.2%	[47]
Total	N/A	6470	4965	1505	23.3%	N/A

*N/A* not applicable

^a^DEL phenotype frequency among RhD-negative Han individuals: 25.5% ± 5.5% (mean ± SD) for the 16 studies, 16.3–32.6% (range)

#### Molecular basis of DEL phenotype among Han

Among 1,266 individuals with molecular data including PCR-SSP (polymerase chain reaction with sequence specific priming) or nucleotide sequencing or both for molecular signals of the *RHD* gene (Table 4), 96.7% were found to carry one distinct variant of the *RHD* gene, designated “Asian type” DEL [49]. The Asian type DEL is also known as RHD*DEL1 and *RHD:c.1227G* > *A* allele [15]. It had originally been described as *RHD*(K409K) in 2001 [9].

**Table 4:** Asian type DEL (RHD*DEL1) in serologic RhD-negative Han who tested positive in adsorption-elution method

Regions	Province/city	RhD-negative individuals (n)		Allele identification (n)						References
				G1227A PCR-SSP		Sequencing		RHD*DEL1 frequency among		
		Total	Adsorption-elution positive	Neg	Pos	RHD*DEL1	Others	Adsorption-elution positive^a^	Rh-negative^b^	
Eastern China	Shanghai	1585	279	11	268	268	11	96.1%	16.9%	[28]
	Shanghai	441	72	4	68	0	4	94.4%	15.4%	[39]
	Shandong	74	22	0	22	ND	ND	100%	29.7%	[40]
Northeastern China	Heilongjiang	374	62	1	61	0	1	98.4%	16.3%	[41]
Southeastern China	Zhejiang, Jiangsu	643	155	0	155	ND	ND	100%	24.1%	[42]
	Jiangsu,Guangdong, Guangxi	165	41	4	37	0	4	90.2%	22.4%	[29]
	Guangdong	102	26	0	26	25	1	96.2%	24.5%	[17]
Central China	Anhui	808	178	10	168	0	10	94.4%	20.8%	[30]
	Anhui	515	142	12	130	0	12	91.5%	25.2%	[43]
	Anhui	155	31	ND	ND	31	0	100%	20.0%	[18]
Taiwan	N/A	395	126	0	126	ND	ND	100%	31.9%	[44]
	N/A	294	94	0	94	94	0	100%	32.0%	[45]
	N/A	118	38	0	38	38	0	100%	32.2%	[47]
Total		5669	1266	42	1193	456	43	96.7%^c^	21.6%^c^	N/A

*ND* not done, *N/A* not applicable

^a^RHD*DEL1 allele frequency among adsorption-elution positive Han individuals: 97.3% ± 3.4% (mean ± SD) for the 13 studies, 90.2–100% (range)

^b^RHD*DEL1 allele frequency among RhD-negative Han individuals: 24.0% ± 5.8% (mean ± SD) for the 13 studies, 15.4%—32.2% (range)

^c^Calculation: (1193 + 31) / 1266 × 100% = 96.7% and (1193 + 31)/5669 × 100% = 21.6%

The Asian type DEL is by far the most prevalent cause of the DEL phenotype in China. Among RhD-negative Han individuals (Table 4), 21.6% of these RhD-negative carried the (molecular) Asian type DEL, closely resembling the 23.3% reported to carry the (serologic) DEL phenotype (Table 3). A similar frequency of 22.0% Asian type DEL was observed among RhD-negative Han individuals who were tested by PCR-SSP only (Table 5).

**Table 5:** Asian type DEL (RHD*DEL1) in serologic RhD-negative among Han by PCR-SSP only

Regions in China	Province/city	RhD-negative individuals (n)	G1227A PCR-SSP (n)		RHD*DEL1 frequency^a^	References
			Negative	Positive		
Southeastern	Zhejiang	143	102	41	28.7%	[101]
Western	Shanxi	2385	1869	516	21.6%	[32, 33]
	Shanxi	30	24	6	20.0%	[102]
Total	N/A	2558	1995	563	22.0%	N/A

^a^RHD*DEL1 allele frequency among RhD-negative Han individuals: 23.4% ± 3.8% (mean ± SD) for the 3 studies, 20.0%—28.7% (range)

*N/A* not applicable

The approach of molecularly testing all serologic RhD-negative individuals in 3 regions (Table 5) corroborated the results from the 13 studies that relied on testing adsorption-elution positive samples only (Table 4). These 13 studies could theoretically have missed some Asian type DEL. They did apparently not miss a clinically relevant number, if any, and the Asian type DEL frequency has firmly been established for the Han population in China.

#### Asian type DEL in other ethnic groups in China

The knowledge of *RHD* alleles is quite limited in RhD-negative individuals of ethnic groups in China other than Han. The first systematic *RHD* allele screen among Chinese was conducted by Qing Wei, Tongji Medical College, Wuhan in 2005 [50]. Dr. Wei examined 50 randomly collected samples from Tibetans, described 4 novel *RHD* alleles and found 1 known variant *RHD* allele. Likely due to the small sample size, all individuals were RhD-positive and no Asian type DEL or other *DEL* allele was detected. Early systematic population screens by other Chinese researchers [51–53] were also productive in discovering novel *RHD* alleles.

Among the 55 other ethnic groups, RhD-negative individuals from only the Hui and Uyghur ethnicities were examined so far (Table 6) in 2 studies [20, 39]. Asian type DEL was found in 17% of the RhD-negative individuals in Hui but only in 2.4% of that in Uyghur (Table 6); the others carrying an *RHD* deletion. The lower prevalence in the Uyghur group are interesting and pointed to the possibility that DEL frequency and *DEL* alleles may vary to some extent among the ethnic groups in China. It will be worthwhile to check a representative set of RhD-negative as well as RhD-positive individuals from each ethnic group to verify their *RHD* alleles. This approach ought to eventually confirm that clinical applications are safe for all ethnic groups in China.

**Table 6:** DEL phenotype and RHD*DEL1 genotype in serologic RhD-negative individuals of ethnic groups in China

Ethnicity	Total RhD-negative individuals (n)	Methods						RHD*DEL1 frequency among		DEL phenotype frequency	References
		Adsorption-elution		G1227A PCR-SSP		Sequencing					
		Neg	Pos	Neg	Pos	RHD*DEL1	Others	Adsorption-elution positive	Rh-negative		
Han	N/A	N/A	N/A	N/A	N/A	N/A	N/A	96.7%^a^	21.7%^b^	23.3%^c^	21 studies^d^
Hui	12	10	2	0	2	2	0	100%	17%	17%	2 studies [20, 39]
Uyghur	127	124	3	0	3	3	0	100%	2.4%	2.4%	2 studies [20, 39]

*N/A* not applicable

^a^See Table 4

^b^Calculation: (1193 + 31 + 563)/(5669 + 2558) × 100% = 21.7%, data from Tables 4 and 5

^c^See Table 3

^d^See Tables 3, 4 and 5. Data shown in this Table 6 for comparison

#### Molecular background of known *DEL* alleles in Han

*DEL* alleles, other than the Asian type DEL, have been determined by nucleotide sequencing in 13 studies [17, 18, 28–30, 39–45, 47]. These studies identified to date a total of 12 additional *DEL* alleles in Han (Table 7) [54–60]. RHD*DEL2, also known as *RHD:c.3G* > *A,* represents the second most frequent *DEL* allele in Han, but at 1.11% it is much less frequent than the Asian type DEL. The remaining *DEL* alleles are even rarer. Of note, 3 of these rare alleles, *RHD(28C* > *T)*, *RHD-CE(4–7)-D* and *RHD-RHCE(10),* were also observed outside of China (Table 7).

**Table 7:** Molecular background of known *DEL* alleles in Han

Allele description	ISBT name	Mechanism	Amino acid changes	Haplotype	Frequency in Han^c^ among		Reports	
					Adsorption-elution positive	Rh-negative	In China	Outside China
*RHD(1227G*>*A)*^a^	RHD*DEL1	Splice site variant	K409K	CDe	96.7%^d^	21.6%^d^	13 studies^d^	3 studies [9, 54, 55]
*RHD(3G*>*A)*	RHD*DEL2	Loss of start codon	M1I	N/R	1.11%	0.25%	4 studies [28, 30, 39, 41]	N/R
*RHD-CE(4–9)-D*	RHD*DEL44	Hybrid allele	hybrid protein	CDe	0.71%	0.16%	3 studies [28, 30, 43]	N/R
*RHD-CE(2–5)-D*	N/A	Hybrid allele	hybrid protein	N/R	0.55%	0.12%	4 studies [28–30, 43]	N/R
*RHD(28C*>*T)*	RHD*01 W.61	Single nucleotide variant	R10W	CDe	0.16%	0.04%	2 studies [28, 39]	GenBank:AM412754
*RHD(53T*>*C)*	RHD*DEL3	Single nucleotide variant	L18P	N/R	0.16%	0.04%	2 studies [28, 39]	N/R
*RHD(251T*>*C)*	RHD*DEL6	Single nucleotide variant	L84P	N/R	0.16%	0.04%	2 studies [28, 39]	N/R
*RHD-CE(4–7)-D*^b^	RHD*01 N.07	Hybrid allele	hybrid protein	cDE	0.16%	0.04%	2 studies [29, 43]	4 studies [9, 55–57]
*RHD(93T*>*A)*	N/A	Single nucleotide variant	F31L	CDe	0.08%	0.02%	1 study [29]	N/R
*RHD(410C*>*A)*	RHD*DEL7	Single nucleotide variant	A137E	CDe	0.08%	0.02%	1 study [28]	N/R
*RHD(838G*>*A)*	RHD*DEL24	Single nucleotide variant	A280T	N/R	0.08%	0.02%	1 study [29]	N/R
*RHD(IVS7*+*152C*>*A,1227G*>*A)*	RHD*DEL36	Splice site variant	K409K	CDe	0.08%	0.02%	1 study [17]	N/R
*RHD-RHCE(10)*	N/A	Hybrid allele	none	CDe	0.08%	0.02%	1 study [28]	2 studies [56, 58]

*N/A* not applicable, *N/R* not reported

^a^Asian type DEL [49]. The report of a genomic deletion of 1013 bp between introns 8 and 9 including exon 9 of *RHD* gene, labelled *RHD(delEx9)*, by Chang et al. in 1998 [59] and Peng et al. in 2003 [60] has not been confirmed and is not considered to be an established basis for any DEL phenotype [10]

^b^Previously descripted as RhD-negative by Wagner et al. in 2001 [9]

^c^Cumulative frequency of *DEL* alleles other than Asian type DEL from 13 studies [17, 18, 28–30, 39–45, 47] (see Table 4)

^d^See Tables 4

#### Serology of DEL phenotype

In 2005, Körmöczi and colleagues [61] suggested that DEL phenotype could be subdivided. They defined a partial DEL phenotype by drawing an analogy to the definition of partial D [62, 63]. In “complete” DEL, the majority of D epitopes were present; in “partial” DEL, the loss of some D epitope was documented [61]. Individuals with partial DEL phenotypes may produce anti-D [61]. According to published data, both the RHD*DEL1 [43] and the RHD*DEL2 alleles [30] lead to a compete DEL phenotype, while hybrid alleles, such as *RHD-CE(4–9)-D* [28, 30, 43], *RHD-CE(4–7)-D* [29, 43] and *RHD-CE(2–5)-D* [28–30, 43], are associated with a partial DEL phenotype [61].

This distinction between partial DEL and complete DEL has major clinical implications: Individuals who carry a partial DEL can develop anti-D when transfused with RhD-positive red cells; individuals who carry a complete DEL cannot develop anti-D.

### Clinical consequences

#### DEL in Chinese transfusion recipients

Mak and colleagues documented in 1993 [34] that anti-D rarely occurred in Hong Kong Chinese and speculated that this could be due to the presence of a very weak form of the D antigen [34]. In 2006, we suggested more specifically [11] that East Asians expressing a DEL phenotype and carrying the RHD*DEL1 allele might not form anti-D after exposure to RhD-positive blood. Wang and colleagues in 2014 [42] found in a retrospective study of 643 RhD-negative patients in China, that 72 pregnant women or transfusion recipients developed anti-D. None of them had a DEL phenotype associated with the RHD*DEL1 allele.

Our literature review documented that transfusion recipients with an Asian type DEL are not known to form anti-D when exposed to RhD-positive blood [42, 64]. Patients with a complete DEL phenotype, such as the RHD*DEL1 allele or Asian type DEL, may safely be managed as RhD-positive with regard to red cell transfusions [11]. Prospective observational studies, however, should monitor this practice [65].

Of course, our proposed strategy cannot be based on DEL phenotypes determined by serologic methods alone [65]. Patients with *DEL* alleles other than the RHD*DEL1 allele, causing the prevalent Asian type DEL, are known to be at risk and can develop allo-anti-D when stimulated by RhD-positive transfusion or pregnancy [61].

#### DEL in Chinese pregnant women

Following the suggestion in 2006 [11] that patients with the RHD*DEL1 allele would not develop anti-D, Shao and colleagues in 2010 [64] investigated 199 RhD-negative pregnant women, 38 of whom (19%) had anti-D. However, 44 other mothers (22%) had an RHD*DEL1 allele, but importantly, none of the pregnant women with the RHD*DEL1 allele had made an anti-D (p < 0.0001; 2 × 2 contingency table, Fisher’s exact test, 2-tailed). Hence, all RhD-negative pregnant women who developed an anti-D lacked the RHD*DEL1 allele. The conclusion of this study had previously been published as a letter [49]: pregnant women with Asian type DEL do not require RhIG (Rh immune globulin) prophylaxis. Similar results were later documented by Wang and colleagues in 2015 [43] who studied the outcome of 142 pregnant women with a DEL phenotype and RhD-positive newborns. Among them, 130 carried the Asian type DEL and did not develop anti-D. In the same report, anti-D was detected after delivery in 6 women with a DEL phenotype. All these 6 women had a partial DEL phenotype caused by *RHD-CE-D* hybrid alleles [43]. In the same year, Xu and colleagues [30] studied 178 pregnant women with a DEL phenotype and RhD-positive fetuses, who declared a history of gestations or birth. Among 176 pregnant women, 168 carried the RHD*DEL1 and 8 the RHD*DEL2 alleles, both being complete DEL, and had formed no anti-D. Anti-D alloimmunization was observed in the remaining 2 women with *RHD-CE-D* hybrid alleles, both being partial DEL. The authors concluded that only pregnant women with *RHD-CE-D* hybrid alleles, representing partial DEL phenotypes, are at risk of forming anti-D [30].

We concur that RhD-negative pregnant women with a complete DEL phenotype, notably the Asian type DEL, do not need RhIG prophylaxis, while pregnant women with a partial DEL phenotype should receive RhIG prophylaxis.

#### DEL in Chinese blood donors

Researchers recognized in 1993 the extremely weak expression of the D antigen by red cell with a DEL phenotype in China and considered it unlikely that transfusion of DEL red cells to RhD-negative individuals could elicit anti-D alloimmunization [34]. Since then, red cells from DEL positive donors have sporadically been reported in single cases from Austria [66], Japan [38, 67] and Korea [68] to induce anti-D. In Han, 1 case of primary and 2 cases of secondary anti-D immunizations in a total of 11 recipients have been reported, who had been transfused with red cells from donors with an Asian type DEL. In 2012, Chen and Liu [69] performed a retrospective study on 104 RhD-negative recipients who received DEL positive red cell transfusion. None of them had developed anti-D [69]. Blood centers have instituted screening blood donors for the *RHD* gene in Germany [12], Switzerland [70], Austria [71], USA [72] and Brazil [73]. None has published this approach to date in China.

Particularly for China, where RhD-negative red cell units are a scarce resource and the DEL phenotype is prevalent, we propose that red cell units from blood donors with any *DEL* allele may be labelled DEL positive, but not RhD-positive. The red cell genotyping for RhD-negative donors needs to be performed only once, for example at first-time donation. Such systematic red cell genotyping would capture the full set of *DEL* alleles in Han individuals and also in the other ethnicities in China, for whom few data are available at this time. The DEL red cell units should still be considered RhD-negative for transfusion purposes, unless the transfusion recipient has formed anti-D before or is currently pregnant. RhD-negative children and women of childbearing age can also be considered.

### Benefits of red cell genotyping for DEL in China

**Fig. 2:**
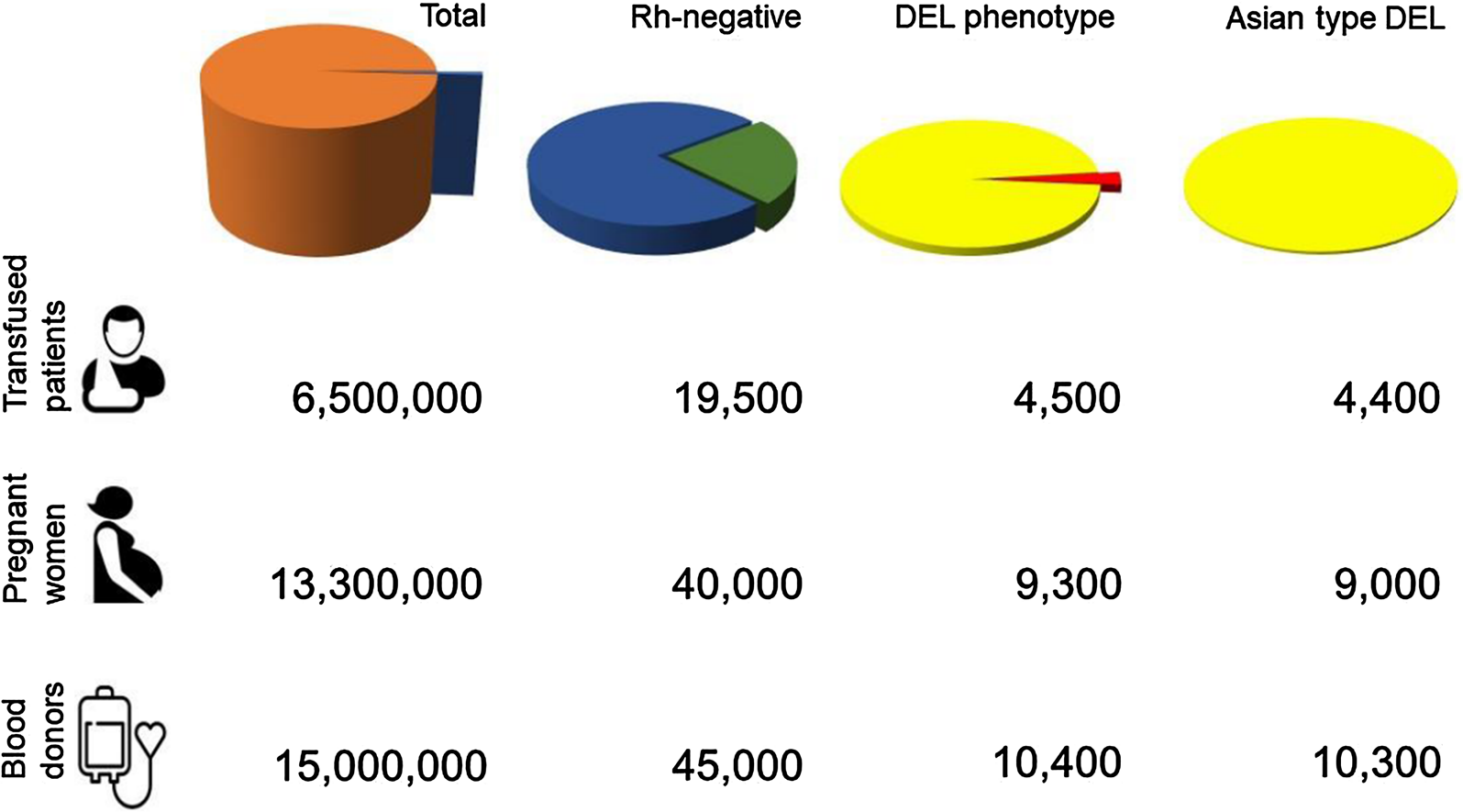
Prevalence of RhD-negative and DEL in clinical cohorts in China per year. The number of individuals with DEL who seek medical care every year, are estimated for the 3 clinical cohorts of transfused patients (upper row), pregnant women (middle row), and blood donors (lower row). Pie charts symbolize the total number (left column), serologic RhD-negative (second column), DEL phenotype (third column), and Asian type DEL (right column). Among the total number of individuals (orange, not to scale), the RhD-negative (blue) represent 0.30% (see Table 2). Approximately 23.3% (see Table 3) of the serologic RhD-negative carry a DEL phenotype (green). The vast majority (96.7%, see Table 4) of all *DEL* alleles (causing a DEL phenotype) is represented by the Asian type DEL (yellow). The small fraction of other *DEL* alleles (red) has a cumulative frequency of 3.3% (see Table 4)

An appreciation of the benefits depends on the number of patients, pregnant women and blood donors involved in China at the national level (Fig. 2). Approximately 19.5 million units of 200 ml red cells were transfused in China in 2013 [74]. Assuming an average of 3 units were transfused to each patient [75], we estimated a total of 6,500,000 recipients per year in China. There were approximately 13.3 million pregnancies in 2019 in China, based on the report of 14.6 million live births and adjusted for multiple birth per pregnancy [76]. The reported number of blood donors was 9.7 million in 2008 in China [77, 78] and we estimated an increase to greater 15 million blood donors per year in 2021 [79].

#### RhD-negative patients

We propose that red cell genotyping for DEL can be performed when a serologic RhD-negative patient is identified, who may require transfusion. Almost a quarter of all RhD-negative patients carry a complete DEL phenotype. The accumulated clinical data in China indicate that these patients could be managed as RhD-positive.

Among the 19,500,000 units red cell units transfused to 6,500,000 recipients [74], an estimated 58,500 units and 19,500 recipients (0.3%) were (serologic) RhD-negative. Also, approximately 13,600 units and 4500 recipients (23.3%) have a DEL phenotype, of which the vast majority carry the Asian type DEL, being a complete DEL phenotype (Fig. 2, upper row). The benefit of an approach to systematic red cell genotyping of all RhD-negative patients is twofold.

First, the transfusion recipients with Asian type DEL will enjoy a much larger supply of red cell units. Any possible delay causing the—unnecessary—procurement of RhD-negative red cells for these patients will be avoided, because an almost unlimited supply of RhD-positive red cells will promptly be available. If blood group antigens, other than the D antigen, need to be matched, because of allo-antibodies, the choice of red cell units for matching is much enlarged. It is known from Western blood supply that patient care is infringed if rare units are needed [80]. Avoiding issues with rare unit supply will hence improve patient care and eventually patient safety.

Second, our proposal will benefit the supply of RhD-negative red cell unit for patients with the definite need for such red cell units [81]. Patients with Asian type DEL will be transfused with RhD-positive red cells. However, red cell units from donors with the Asian type DEL or any other *DEL* alleles are still contributing to the pool of RhD-negative red cell units for transfusion. Hence, the supply of RhD-negative red cell units for truly RhD-negative recipients would be increased by 30% (calculation: 0.23/0.77 = 0.299).

#### RhD-negative pregnant women

Red cell genotyping for DEL can be performed once a pregnancy is recognized in a serologic RhD-negative woman. Pregnant women with a complete DEL phenotype, do not need RhIG prophylaxis. Pregnant women with a partial DEL phenotype should still receive RhIG prophylaxis.

Of the 13.3 million pregnancies per year [76], approximately 40,000 were expected to occur in (serologic) RhD-negative women. Approximately 9300 (23.3%) of them will have a DEL phenotype, of which 9000 (96.7%) carry a complete DEL phenotype, represented by the RHD*DEL1 or RHD*DEL2 alleles (Fig. 2, middle row).

At present, most pregnant women with RhD-negative in the USA will receive at least one antepartum injection of RhIG and a quarter of them may receive a second injection [81]. RhIG may be unavailable or not regularly obtained at public hospitals on mainland China, although it can occasionally be sourced through select international medical facilities at extra expense [82]. Some RhD-negative pregnant women may need to cover the costs without reimbursement from insurance [82]. RhIG from private hospitals in big cities like Shanghai can cost 700 US$ per injection or from pharmacies in Hong Kong 300 US$ per injection [83].

The positive cost–benefit of using red cell genotyping to guide RhIG prophylaxis among pregnant women with RhD-negative phenotype has been described for the USA [84]. The cost–benefit in China is much greater as 21.6% (Table 4) of the RhIG doses can be saved compared to less than 5% in the USA [81, 84–86]. For China, if red cell genotyping for *DEL* alleles were performed in RhD-negative women with a complete DEL phenotype, approximately 18,000 doses of RhIG annually can be avoided and hence up to 12,600,000 US$ can be saved annually.

#### RhD-negative blood donors

We propose to explore if red cell genotyping for DEL can routinely be performed in donors. Red cell units from blood donors with any *DEL* allele should be labelled DEL positive. Such DEL red cell units can still be considered RhD-negative for most transfusion purposes.

Of the 12 million blood donors in China per year, approximately 36,000 are expected to be (serologic) RhD-negative. We estimated that approximately 8300 donors (23.3%) have a DEL phenotype, of which the vast majority carry the Asian type DEL (Fig. 2, lower row).

An estimated 1.7 million individuals in China carry the DEL phenotype. If these individuals could be explored as potential DEL donors to establish a separate DEL donor pool, this strategy would significantly lessen the demand for RhD-negative blood and would have a profound effect on the supply of red cell units for transfusion to RhD-negative recipients.

### Practical strategy for resolving RhD-negative individuals with DEL phenotype

We propose that red cell genotyping for DEL can be performed whenever a (serologic) RhD-negative phenotype is detected in potential transfusion recipients, pregnant women and blood donors. Recipients and pregnant women with a complete DEL phenotype, notably the Asian type DEL, should be managed as RhD-positive with regard to transfusion or RhIG administration or both. Blood donors with any DEL phenotype can still be considered RhD-negative for most transfusion purposes. Systematic red cell genotyping has often been reviewed [87–96], beginning in 2001 for clinical applications [12] and red cell unit management [97, 98].

#### Algorithm

**Fig. 3:**
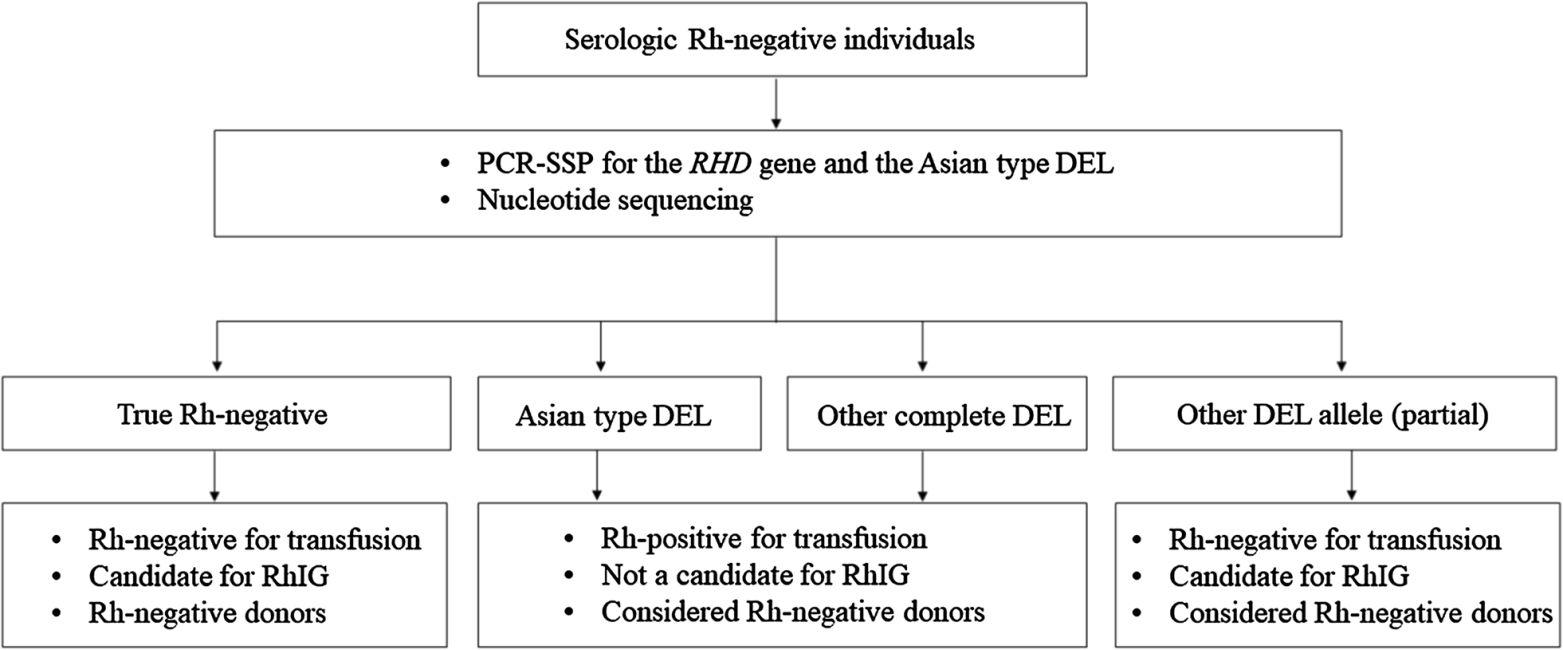
Algorithm to resolve a serologic RhD-negative phenotype by red cell genotyping for DEL. All individuals in the 3 clinical cohorts (see Fig. 1) can be tested for the presence of the *RHD* gene and the Asian type DEL. Typically, rapid screening, such as PCR-SSP assays, can resolve the Rh type. Only in rare cases, nucleotide sequencing will be required, if resolving other complete DEL and partial DEL is desired

To facilitate implementation of our proposals, we developed an algorithm to resolve red cell genotyping results among serologic RhD-negative individuals (Fig. 3). PCR-SSP of *RHD* intron 4 and exon 7 had a greater positive predictive value for the presence of *RHD* gene [9, 88] than screening for other exons, such as *RHD* exon 10 [99, 100]. PCR-SSP of the *RHD*(1227G > A) single nucleotide variant (SNV) in exon 9 has successfully been applied to detect Asian type DEL in many studies [9, 17, 18, 28, 29, 32, 33, 39–42, 44, 45, 47, 88, 101–106].

Complete *DEL* alleles, other than the Asian type DEL, and all partial *DEL* alleles can be specifically detected by nucleotide sequencing [9], if desired. A need for nucleotide sequencing would occur in less than 4% of all RhD-negative individuals (Table 4). Nucleotide sequencing can, however, be particularly informative in ethnic groups, other than Han, where the DEL frequency and alleles may vary from the known situation in Han and remains currently largely unexplored.

#### Cost-effectiveness of red cell genotyping

Eventually, manufacturers of *RHD* genotyping assays and systems may offer cost-effective red cell genotyping tests designed to identify the presence of the *RHD* gene and the Asian type DEL. Our literature review documented that the accuracy to identify Asian type DEL in Han by PCR-SSP is at least comparable to adsorption-elution methods and similar to nucleotide sequencing.

Kacker and colleagues [84] evaluated the financial implication of *RHD* genotyping to guide RhIG prophylaxis for pregnant females in the USA and found a saving for the medical care system at any cost of 256 US$ or below. The national insurance system in China reimburses 80–90% of the cost if the medical service is approved by the China Ministry of Health, while most genetic tests, including any red cell genotyping, are currently not approved and thus not paid by the insurance [107]. A cost-effectiveness analysis may be performed, as in other health care systems before [84], and be used to develop recommendations in a commissioned report by the government or in a ‘white paper’.

#### Complexity of red cell genotyping for DEL

Red cell genotyping in general and DEL screening in particular are not widely available and accessible in China. Currently, molecular DEL typing is mainly performed through the clinical laboratories affiliated with few top-ranked hospitals in major cities such as Shenzhen [17], Shanghai [28], Hefei [18], Nanjing [29], and similar institutions. Most local hospital-affiliated laboratories may not be outfitted for such test. Although DEL molecular typing could also be offered as part of a research program by universities and other institutions, no such diagnostic result reported by research laboratories is generally authorized for clinical care [107]. The increased market demands have stimulated development of private commercial laboratories, where advanced hardware and data management could be harnessed for DEL molecular testing. Medical genetic professionals should be trained in this precision medicine application and could be employed much wider than in a few public hospitals. Presently, samples for red cell genotyping, such as DEL molecular testing, can be sent to reference laboratories, where standardization of laboratory methods is promoted and the economy of large-scale testing can be achieved [81].

## Conclusion

The DEL phenotype is a D variant with low amounts of D antigens on the red cell surface occurring among RhD-negative individuals, who are rare in East Asia. The prevalence of DEL and its molecular bases are well characterized for the Han population, whereas sufficient information for other ethnic groups in China is lacking. Red cell genotyping for DEL is predicted to lessen the demand for RhD-negative blood, save money for RhIG injection and increase the pool of red cell donors available for RhD-negative transfusion. The distinction of complete DEL and partial DEL is required and can—as a practical routine approach—only be achieved by molecular methods. We propose to explore a timely introduction and full integration of molecular typing for DEL into the clinical practice of China.

## Statement of Disclaimer

The views expressed do not necessarily represent the view of the National Institutes of Health, the Department of Health and Human Services, or the U.S. Federal Government.

## Data Availability

Not applicable.
